# The Two-Phase Emergence of Non Pandemic HIV-1 Group O in Cameroon

**DOI:** 10.1371/journal.ppat.1005029

**Published:** 2015-08-04

**Authors:** Marie Leoz, Felix Feyertag, Anfumbom Kfutwah, Philippe Mauclère, Guillaume Lachenal, Florence Damond, Fabienne De Oliveira, Véronique Lemée, François Simon, David L Robertson, Jean-Christophe Plantier

**Affiliations:** 1 Laboratoire de Virologie, CHU Charles Nicolle, Rouen, France; 2 EA 2656 GRAM, Université de Rouen, Rouen, France; 3 Computational and Evolutionary Biology, Faculty of Life Sciences, University of Manchester, Manchester, United Kingdom; 4 Service de Virologie, Centre Pasteur du Cameroun, Yaoundé, Cameroun; 5 Direction Interarmées du Service de Santé, Nouméa, Nouvelle Calédonie; 6 Laboratoire SPHERE, UMR 7219, Université Paris Diderot & Institut Universitaire de France, Paris, France; 7 Service de Virologie, APHP CHU Bichat Claude Bernard, Faculté de Médecine Paris Diderot, Paris, France; 8 Laboratoire associé au Centre National de Référence du VIH, CHU Charles Nicolle, Rouen, France; 9 Service de Microbiologie, APHP CHU Saint Louis, Faculté de Médecine Paris Diderot, Paris, France; University of Glasgow, UNITED KINGDOM

## Abstract

Unlike the pandemic form of HIV-1 (group M), group O viruses are endemic in west central Africa, especially in Cameroon. However, little is known about group O’s genetic evolution, and why this highly divergent lineage has not become pandemic. Using a unique and large set of group O sequences from samples collected from 1987 to 2012, we find that this lineage has evolved in successive slow and fast phases of diversification, with a most recent common ancestor estimated to have existed around 1930 (1914–1944). The most rapid periods of diversification occurred in the 1950s and in the 1980s, and could be linked to favourable epidemiological contexts in Cameroon. Group O genetic diversity reflects this two-phase evolution, with two distinct populations potentially having different viral properties. The currently predominant viral population emerged in the 1980s, from an ancient population which had first developed in the 1950s, and is characterized by higher growth and evolutionary rates, and the natural presence of the Y181C resistance mutation, thought to confer a phenotypic advantage. Our findings show that although this evolutionary pattern is specific to HIV-1 group O, it paralleled the early spread of HIV-1 group M in the Democratic Republic of Congo. Both viral lineages are likely to have benefited from similar epidemiological contexts. The relative role of virological and social factors in the distinct epidemic histories of HIV-1 group O and M needs to be reassessed.

## Author Summary

HIV-1 group O is one of the causal agents of AIDS, together with HIV-1 groups M (responsible for the pandemic), N and P (15 and 2 cases detected respectively, from Cameroon) and HIV-2 groups A to I (mostly found in West Africa), each group resulting from a distinct cross species transmission event from non-human primates. Even though mostly restricted to Cameroon, group O infections have been found in other African countries as well as in Europe and in the US. Due to their genetic distance from the pandemic HIV-1 group M, group O viruses still impact diagnosis, virological and therapeutic monitoring. Moreover, very few data are available on the natural history and epidemiology of these infections, as well as their genetic diversity and evolution. In particular, there is currently no explanation of the lack of spread of these variants, compared to the pandemic viruses from group M. Analysis of HIV-1 group O molecular evolution, from sequences spanning more than 2 decades, is an opportunity to better understand the phylodynamics of group O infection. We investigate it further by producing the largest set of group O sequences described. We show that the previous classifications proposed do not agree with each other and do not fit with the extensive genetic diversity of this group. We also estimate that group O MRCA existed in the 1930s (95% Higher Posterior Density: 1914–1944), and show that group O has diversified during two successive phases that could be linked to the specific historical context of Cameroon. These results contribute to a better understanding of the factors influencing HIV evolution, especially in the local context of west central Africa and lead to new hypotheses on the limited diffusion of such variants.

## Introduction

Human Immunodeficiency Virus Type 1 (HIV-1) is comprised of four groups (M to P), each originating from a distinct cross species transmission event from Simian Immunodeficiency Virus (SIV) variants circulating in apes [1,2,3]. The major group (M) has spread worldwide from Central Africa during the second part of the 20^th^ century [4,5], while groups N and P are extremely rare. These latter two groups have arisen more recently and have only been identified so far in 15 and 2 patients respectively [1], all but one from Cameroon. Finally, despite group O’s origin being estimated to be about the same time period as group M [6], the group O (outlier) epidemic is mostly restricted to Cameroon, and has remained stable since the 1990s, whereas group M has been spreading dramatically [7,8,9].

Little is known about natural history of group O infection, but the limited follow-up data available [7,10] indicate that, as with group M, horizontal as well as vertical transmission contributes to its spread, and untreated patients show high plasma viral load, leading to a loss of CD4 T cells and eventual progression to AIDS. Thus, the natural history of group O infection seems to be similar to that of group M, even though some studies have shown distinct virological properties such as a lower replication capacity [11,12] or failure to counteract some cellular restriction factors [13,14].

There is high genetic distance between M and O strains, with 67%, 73% and 56% sequence identity between group M HXB2 [15] and O ANT70 [16] prototype strains in *gag*, *pol*, and *env* genes, respectively. As a result, diagnosis and follow-up of group O infections require adapted tools [1]. Group O natural polymorphism also has an impact on treatment options, since most strains naturally present the Y181C mutation in the Reverse Transcriptase (RT) conferring resistance to Efavirenz and Nevirapine (first generation Non Nucleoside RT Inhibitors, NNRTIs). Of particular note, these molecules are part of the most common first line antiretroviral therapy combinations used in Cameroon. Taken together, these group O characteristics can lead to delayed diagnosis, underestimated viral loads or treatment failure, if the nature of the group O infection status is not taken into account.

Not only is group O highly distant from group M, but over nearly a century since its introduction into the human population, a high level of intra-group genetic diversity has developed and several attempts have been made to characterize it. Different classifications have been proposed, and these have defined 3 clades [17,18], 5 clusters [19] and more recently, only 2 lineages (C181 or Y181) based on the residue at the RT position 181 [20]. The relevance of these different classifications, which were based on few sequences and never compared to each other, needs to be evaluated. More importantly, the processes that led to this apparent complexity are still to be understood.

No significant group O epidemic has been described outside Cameroon, and only sporadic cases appear in other African countries (mostly west central Africa), in the US and in Europe [1]. In France, the RES-O survey network has identified 143 patients infected with group O since 1992 [10,21], the largest series of group O infections identified outside of Cameroon. Most of these patients originate or are linked to patients originating from Cameroon, due to historical links between France and this region.

This absence of epidemic spread outside of the Cameroon is surprising, since group O infections have been identified on three different continents, as far back as 20 years ago in North America and 50 years ago in Western Europe, and an estimated presence close to a century old in Africa. Interestingly, it has recently been proposed that group M expansion in the capital of the Democratic Republic of Congo (DRC) Kinshasa had benefited from different contextual changes in the 1960s, leading to a dramatic increase of the number of infections at that time [5]. The authors did not observe such a change in group O growth rate, thus hypothesising that this explained the different epidemiological histories of groups M and O.

Here we have used the largest set of HIV-1 group O sequences assembled to date, obtained from 190 patients sampled in France or in Cameroon on a time scale of 26 years, to better understand group O emergence and evolution, by investigating the dynamics of their diversification and its consequences.

## Results

**Fig 1:**
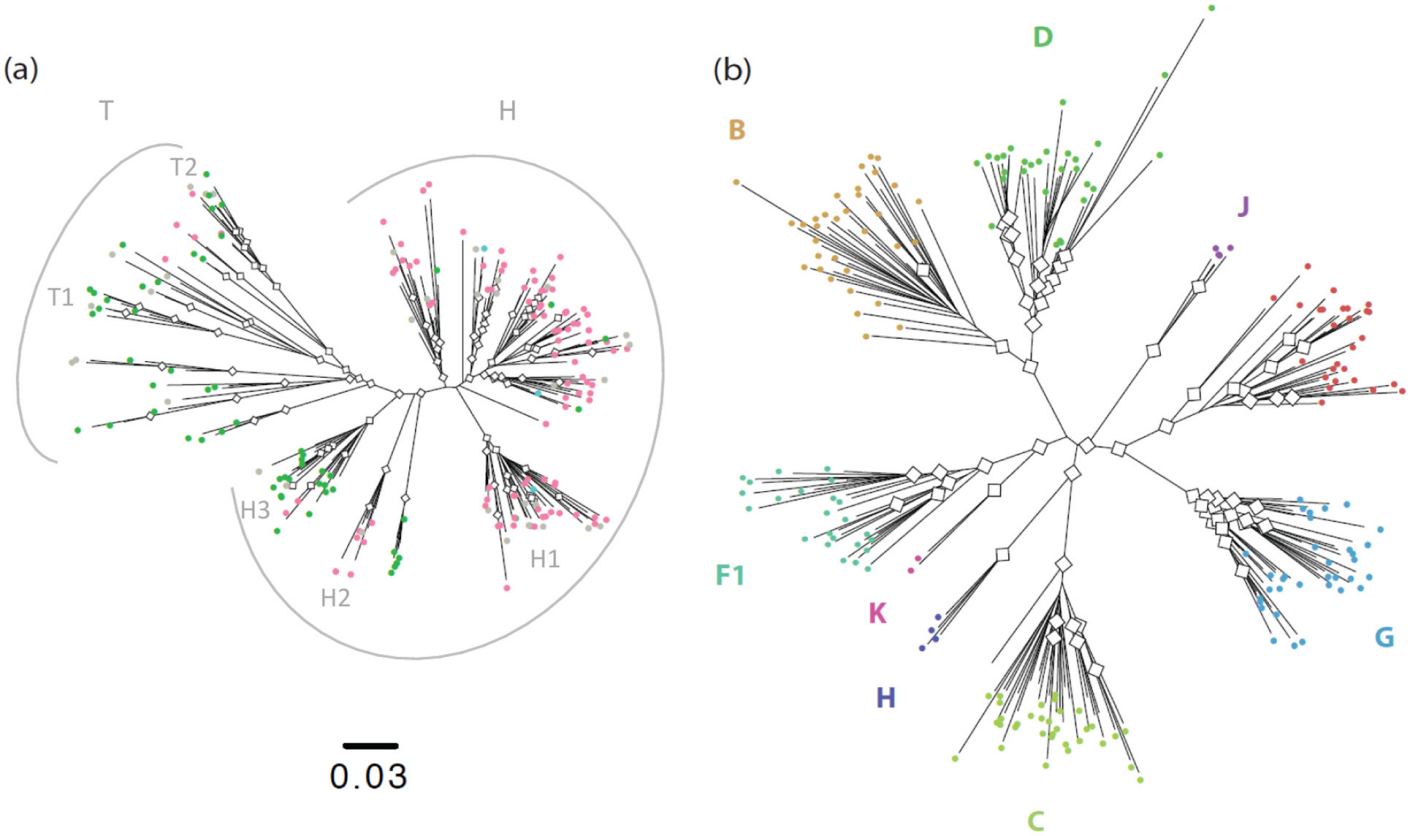
Phylogenetic analysis of HIV-1 sequences. **a)** Maximum likelihood tree inferred from the 190 concatenated group O sequences, with white diamonds at nodes with bootstrap values >70 and colours highlighting the nature of the residue at position 181 in the Reverse Transcriptase: Pink = C (N = 116); Green = Y (N = 71); Blue = mixed Y and C (N = 3); Grey = sequences from patients non NNRTI-naïve or with no data about NNRTI treatment. **b)** Maximum likelihood tree inferred from the 190 concatenated group M sequences.

The maximum likelihood tree obtained from 190 concatenated sequences showed that a large proportion of the strains fall in a major subgroup. The short branch lengths in this major subgroup when compared to the long ones in the minor subgroup (S1 Text and S1 Fig) give the tree a comet-like shape (Fig 1A), as opposed to the well-defined double star structure of the pandemic group M subtypes (Fig 1B). Due to this particular topology, we defined the major subgroup (N = 147) as corresponding to the "comet head" or H strains, and the minor subgroup (N = 43), as corresponding to the "comet tail" or T strains. Subclusters could be observed among the H strains (H1, H2 and H3) and the T strains (T1 and T2). This classification encompasses the previous ones that were partially discordant (see S2 Text and S2A and S2B Fig).

**Fig 2:**
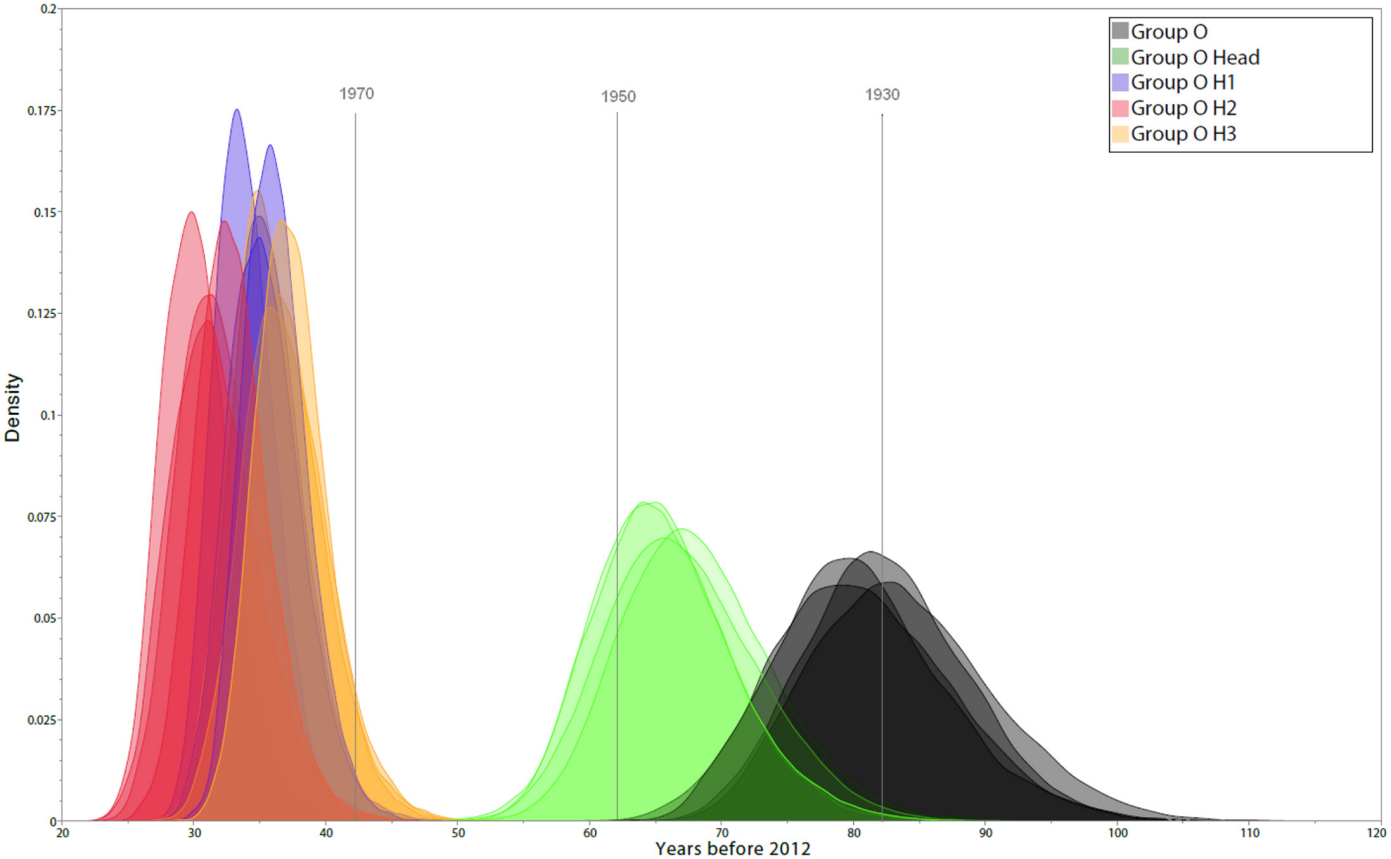
Estimates of group O tMRCA. The marginal posterior density curves obtained using four different population growth models are shown. Different colours identify the distribution of tMRCA estimates for global group O (black), subgroup H (green), and subclusters H1, H2 and H3 (blue, red and yellow, respectively).

**Fig 3:**
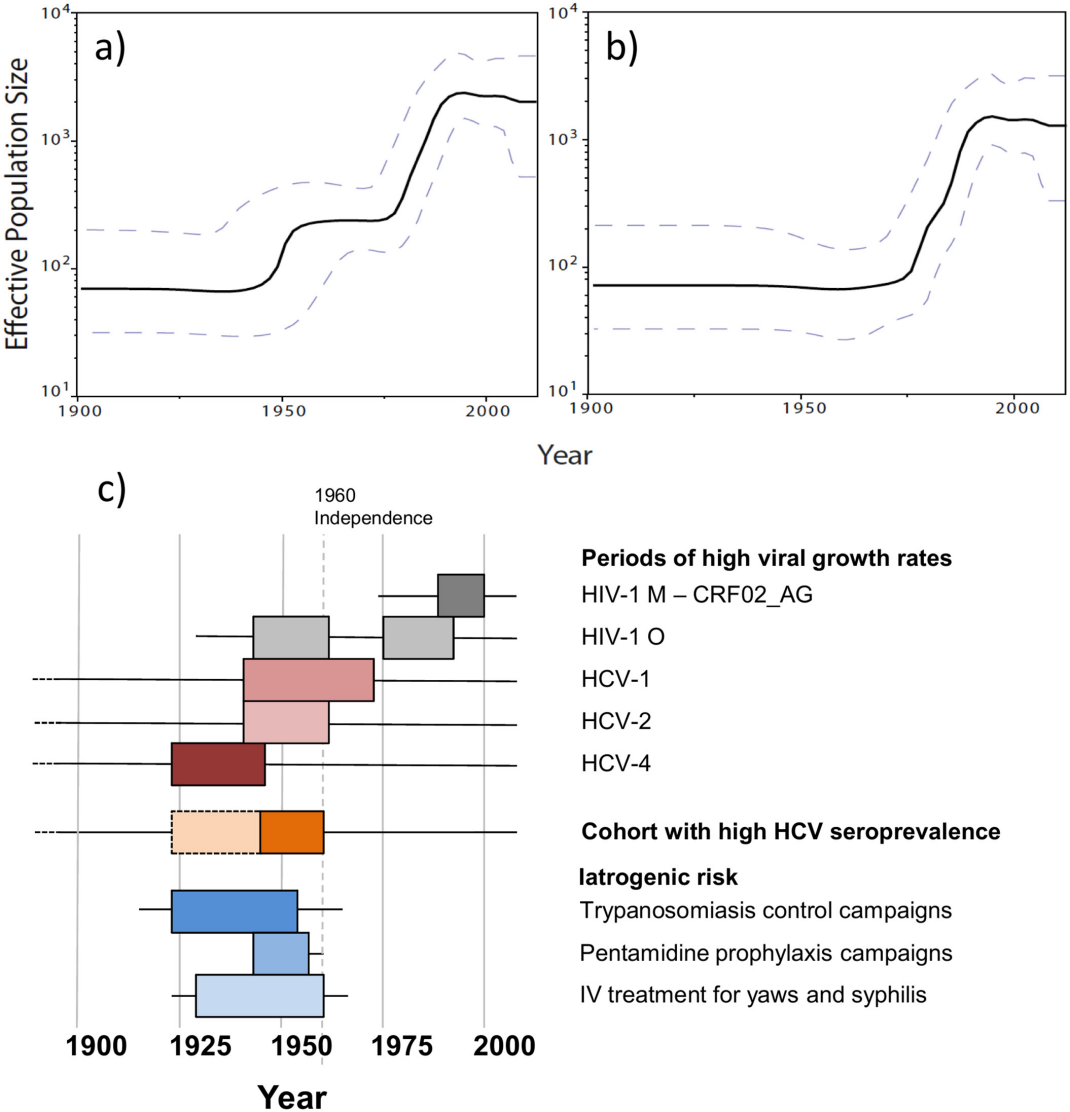
Dynamics of group O populations over time and contemporaneous contextual elements in Cameroon. **a)** Bayesian Skyline Plot (BSP) inferred from the 154 concatenated group O sequences for which the three genome regions were obtained from a single time-point sample. **b)** BSP inferred from a subset of these sequences (N = 137), including the H strains (N = 124) and clusters T1 (N = 6) and T2 (N = 7) from the T strains. **c)** Periods of high viral growth rates for HIV-1 group M CRF02_AG [22] (dark gray box), HIV-1 group O (grey boxes), HCV-1 [23] (dark pink box), HCV-2 [23] (pink box) and HCV-4 [23] (brown box) in Cameroon; cohort with high HCV seroprevalence in Cameroon [24] (orange box); population-scaled risk factors for iatrogenic transmission during trypanosomiaisis control campaigns in rural Cameroon [25] (peak period: dark blue box), pentamidine prophylaxis campaigns in rural Cameroon [25,26] (blue box) and intra-venous treatment for yaws and syphilis in Cameroon [25] (peak period: light blue box).

We used 154 sequences for which all three gene fragments were obtained from a single sample of known sampling time to investigate group O origins and dynamics over time. Different coalescent population growth models (constant size, exponential growth, logistic growth and Bayesian skyline) gave consistent estimates for group O’s time to most recent common ancestor (tMRCA) of around 1930 (Fig 2, black curves), with 95% highest posterior densities (95% HPD) ranging from 1914 to 1944. Interestingly, Bayesian Skyline Plots showed that group O genetic diversity had gone through an alternation of slow and fast growth phases (Fig 3A). Two waves of exponential growth were observed, the first around 1950 and the second, longer and more important, starting in the late 1970's and ending in the early 1990s. While the first wave could be observed when investigating all of the 154 sequences (Fig 3A), it did not appear when only including the H strains and the two minor subclusters observed among the T strains, T1 and T2 (Fig 3B). These results indicate that the first wave represents the development of an ancestral level of genetic diversity, and the second wave the emergence of subpopulations such as H strains.

We then investigated the date of subgroup H MRCA using the same 4 growth models, which consistently gave an MRCA estimated around 1945 (best fitting model 95% HPD: 1933–1955) (Fig 2, green curves). This suggests that H strains MRCA was present among the background of genetic diversity that had arisen during the first exponential growth phase in the 1950s. Among these H strains, we observed some important and strongly supported subclusters H1, H2, H3, which all together represented 34% of the group O strains studied herein. Their respective MRCA estimates were even more recent, all being estimated to have appeared between 1975 and 1980 (Fig 2, blue, red and yellow curves), the very beginning of the second exponential growth phase.

When studying the distribution of the Y181C resistance mutation among the 100 samples collected from NNRTI-naïve patients (77 H strains, 23 T strains), we observed that this profile was naturally present in 65 strains (65%), 62 of which were H strains and only 3 T strains. Thus, 80.5% of the H strains naturally presented the resistance mutation and only 13% of the T strains, indicating a strong association between subgroup H and C at position 181 (Chi-squared test: p<10E-5). Moreover, among the 15 H strains presenting a 181Y residue, 11 (73.3%) belonged to a single subcluster (H3).

Bayesian analyses indicated that the mean evolutionary rate of H strains was significantly higher than that of the T strains (Student T test: p<10E-5; see Table 1). For both subgroups, the dN/dS ratios indicated a globally negative selection pressure on the genome regions investigated, with this ratio was also significantly higher in subgroup H than in subgroup T (Likelihood Ratio Test: p = 7.10E-4, see Table 1). Thus, H strains evolved at a higher rate and had lower negative selection pressure than T strains. Evolutionary analyses also indicated that the mean growth rate of subgroup H was significantly higher than that of the T (Student T test: p<10E-5; see Table 1), consistent with the apparent predominance of the H strains.

**Table 1:** Comparison of rate and ratio estimates between H and T subgroups.

		Mean*	95% HPD^$^ / 95%CI^£^	p^#^
Evolutionary rate	H	1,50E-03	[1,21E-03; 1,80E-03]	<10E-05
	T	1,14E-03	[8,64E-04; 1,44E-03]	
Growth rate	H	1,20E-01	[9,38E-02; 1,47E-01]	<10E-05
	T	6,14E-02	[3,68E-02; 8,69E-02]	
dN/dS	H	2,94E-01	[2,82E-01; 3,06E-01]	7,40E-04
	T	2,43E-01	[2,29E-01; 2,58E-01]	

* the mean value for evolutionary and growth rates were estimated using BEAST, under the best fitting model (GTR+Γ+I and exponential growth); the mean values for the Non Synonymous (dN) versus synonymous (dS) mutation ratios were estimated using HyPhy under the GTR model.

^$^ the 95% Higher Posterior Densities (HPD) upper and lower bound for evolutionary and growth rates were estimated using BEAST, under the best fitting model (GTR+Γ+I and exponential growth).

^£^ the 95% confidence intervals (CI) for the Non Synonymous (dN) versus synonymous (dS) mutation ratios were estimated using HyPhy under the GTR model.

^#^ the p-value for the difference between two means was calculated using student T test; the Likelihood Ratio Test p-value against the hypothesis of a single rate was estimated using HyPhy under the GTR model.

## Discussion

Even though both HIV-1 groups O and M’s MRCAs have been estimated in the early 20^th^ century, group O, unlike group M, has not spread globally and remains endemic in Cameroon. The causes of this are not fully understood: unlike HIV-1 groups N and P, group O has been successful enough to infect tens to hundreds of thousands people [3,18] and no evidence of lower transmissibility has been shown *in vivo*, as it has been for HIV-2 [27]. Thus, aside from potential intrinsic properties [11,12], epidemiological and related contextual factors could also have played a role in this difference between the two main HIV-1 lineages [5].

Our data confirmed that group O diversification represents a continuum of diversity consistent with local diversification in a geographically restricted area (see S2 and S3 Texts and S2 Fig), while the group M subtypes are mostly the result of founder effects following the introduction of single strains to dispersed geographic locations [28]. We also confirmed the existence of a predominant population (H strains—from the comet Head), which was strongly associated to the C mutation at the Reverse Transcriptase position 181, but our data demonstrated that this mutation alone is not an adequate marker for classification as proposed by Tebit *et al*. [20]. Indeed, it was not possible to just split the tree in two C181 and Y181 subpopulations, even if hypothesizing unlikely transmissions of strains with acquired mutations for the few T strains harbouring this Y181C mutation.

The existence of this major subgroup raised questions on the evolutionary processes that could have shaped the particular tree topology. Our estimation of group O MRCA around 1930 (95% HPD: 1914–1944) is close to the previous estimates of 1920 [6] which was based on fewer sequences. This result confirms that group O MRCA was contemporaneous to that of group M, estimated in the beginning of the 20^th^ century [4]. But unlike recent conclusions of a constant low growth rate for group O by Faria et al. [5], we show two phases of exponential growth, the first during the 1940–1960 time window and the second one during 1970–1990.

The causes for these two phases are probably complex and multifactorial. However, the increase in growth rates around 1950 coincides with a period of high-rate of transmission of HCV in Cameroon, as shown in both epidemiological [24] and phylogenetic studies [23], (Fig 3C). In Southern Cameroon, HCV infection is a reliable marker of iatrogenic transmission, associated in elderly people with a history of exposure to medical campaigns and interventions involving unsterile procedures [29]. The 1940–1960 period saw a rise in injection practices globally [30]. Colonial medical activity in Cameroon peaked during this period, and was dismantled after independence (1960) for political and epidemiological reasons (low incidence of sleeping sickness and yaws) [25]. Since the first plateau identified in this study matches the decline of HCV transmission in Cameroon after 1960, the first phase of group O rapid growth can thus reflect a scenario of iatrogenic amplification following an event of cross-species transmission, as proposed for HIV-1 M in the Congo basin region [31,32]. The booming city of Douala may have been a favorable epidemic context—combining iatrogenic and socio-sexual factors—for the initial diversification of group O. Indeed, this is most probably where a visiting Norwegian sailor—the first reported case of group O infection—became infected in 1962 [33,34], suggesting that the virus was established in this city by the early 1960s.

Two decades later, the greater and longer second phase of exponential growth started in the late 1970's and reached a plateau in the early 1990s. Though group O might have found a favourable social context for transmission during this period, urban growth per se cannot explain the exponential increase in new infections, since rates of urban growth in Yaoundé and Douala were maximal in the post-second war years (at about 10%) and have declined since, down to 5–7% in the 2000’s [35,36,37,38]. Further investigations are thus needed to understand this second phase of diversification. Intrinsic viral properties could have been involved, since the second wave of diversification is linked to the development of several viral subpopulations, some minor in the T subgroup (T1 and T2), and the H strains which became predominant. Of note, subclusters H1, H2 and H3, which MRCA was estimated to be no older than 1975, now represent 34% of the strains included in this study. They have been sampled either in Cameroon or in France, at time points ranging from 1994 to 2012, showing that they are not associated to a particular sampling time or location (see S2 Text and S2C and S2D Fig). Selection pressure analysis also showed that H strains were submitted to lower negative selection pressure than T strains and evolved faster. It has also been proposed that the 181C residue in the RT could represent a fitness advantage [20], even though this has been demonstrated *in vitro* only using mutants viruses from a H strain backbone. The reasons why H strains became predominant could thus be linked to favourable biological properties of these strains and/or different opportunities for diffusion. The two phases may also reflect a two-step geographic expansion of the virus in Cameroon, but the absence of a geolocalized dataset does not allow us to investigate this hypothesis further.

Our data thus show that group O genetic diversity and phylogenetic topology are due to their evolution in alternating slow and fast phases that could be related to specific events in the history of Cameroon, contrasting with recent conclusions [5]. However, these variants failed to become pandemic despite two exponential growth phases. Interestingly, the end of the last exponential phase coincides with the time of introduction and spread of CRF02_AG, the predominant group M form in Cameroon, after it originated in the 1970's in the DRC [22] (Fig 3C). This led to the previous observation in Cameroon of a drop in group O infections among HIV-1 positive samples in the early 1990s, when the M group rose exponentially while group O remained stable or even slightly decreased [8,9]. How group M became predominant over the group O epidemic in Cameroon could be due to different virological properties [11,12] and/or a competition between the two epidemics, as suggested for HIV-2 in West Africa [39]. This would also explain the absence of French group O epidemic (see our phylogeographic analyses in S2 Text and S2C Fig), contrasting with HIV-1 group M molecular epidemiology. Most of the 143 group O patients identified originate from Cameroon, while after the long predominance of group M subtype B in France, non-B strains have been imported from sub-Saharan Africa and now circulate in patients from various risk groups [40]. Thus, the absence of diffusion of group O in France has to be explained by other factors than a lack of boundaries between different epidemiological populations.

In summary, our results on group O genetic diversity support the conclusion that it cannot be divided into subtypes similar to group M, but a major subgroup (H) emerged from a genetically diverse minor subgroup (T). Contrary to Faria's findings [5], these two populations reflect the fact that group O underwent two waves of rapid diversification in the 20^th^ century. Although this evolutionary pattern is specific to group O, the HIV-1 group O epidemic in Cameroon paralleled the spread of HIV-1 group M in the DRC. This is certainly linked to similar factors as those described for group M, such as iatrogenic amplification and the favorable urban context of fast-growing and cosmopolitan cities. In this light, the emergence of group M in Kinshasa may be seen as unexceptional, however expansion of groups O and M occurred in distinct epidemiological and socio-demographic contexts in Cameroon and the DRC respectively, which could have led to their different epidemiological patterns [2].

While our study reveals important information about HIV-1 group O’s emergence and evolution, investigations are still needed to understand the other reasons for their unsuccessful spread compared to that of group M—as well as for group O T strains compared to that of H strains—especially by exploring the biological properties of these divergent viruses.

## Materials and Methods

### Patients and samples

Samples from 190 patients were included in the analysis, 102 of which were sampled in France, 87 in Cameroon, and 1 in Gabon. The time of sampling spanned from June 1987 to February 2012, but was undetermined for 4 samples.

In France, the samples were collected from hospitals located all across the country by the RES-O survey of the French HIV National Reference Centre in Rouen. In Cameroon, samples were collected at the Centre Pasteur du Cameroun from different parts of the country: Centre (N = 25), Littoral (N = 7), North (N = 4), and South (N = 1) regions. For 50 samples, the collection site in Cameroon was not determined. The sample from Gabon was collected in Libreville.

The nature of the samples analyzed was plasma or serum (N = 173), PBMCs (N = 6) or supernatant from viral culture (N = 11).

### Ethics statement

The viral sequences we analyzed were produced from leftover samples that had previously been collected for routine diagnosis or follow up of the patients. Thus, no additional sample was performed specifically for this study, and we did not use any information about the patients from whom this samples had been obtained. As a consequence, no consent from the patients nor approvement from ethics committee was requested.

### Genome amplification and sequencing

Three fragments from two genes (prRT: *pol* protease and partial RT, 987 bp; int: *pol* partial integrase, 603 bp, gp41: *env* partial gp41, 522 bp) were amplified by nested PCR or RT-PCR, depending on the sample type, and sequenced as previously described [41]. Accession Numbers: KT198045—KT198614.

### Genetic diversity

#### Phylogenetic analyses

Sequences of three gene fragments from the 190 strains were codon-aligned using MUSCLE in MEGA 5.0 [42]. In *en*v gp41, a small hypervariable region with insertions, corresponding to the loop between HR1 and HR2, could not be unambiguously aligned and was thus removed. Maximum likelihood phylogenetic trees were inferred from each of the three alignments, as well as from a concatenated alignment of the three regions, using RAxML [43] rapid hill-climbing algorithm on randomized stepwise addition parsimony trees. A General Time Reversible model with Gamma-distributed evolutionary rates among sites (GTR+Γ) was used to compute 200 candidate ML phylogenetic trees for each gene alignment. Of these, the tree with the highest likelihood was selected, and a 1000-replicate parametric bootstrap analysis was performed to assess the reliability of branching order. The ML trees were visualised using FigTree (http://tree.bio.ed.ac.uk/software/figtree/).

#### Distribution of the natural Y181C mutation

Among the 190 concatenated sequences, 11 were sampled from patients who received or had received NNRTIs and 100 were NNRTI-naïve at the time of sampling. The information about NNRTI treatment was not available for the other 79 patients. To investigate the distribution of the natural 181C resistance mutation, we used the sequences from the 100 NNRTI-naïve patients and studied the association between clade and Y181C residue by using a chi-squared test.

#### Evolutionary analyses

Out of the 190 concatenated sequences described above, 154 were used for tMRCA inference and analysis of evolutionary dynamics. These were the sequences for which all three regions had been sequenced from the same sample, meaning a unique time of sampling. All the positions in the protease and RT regions known to be involved in drug resistance for group M according to the ANRS algorithm (http://www.hivfrenchresistance.org/) were removed from the alignment. SIV sequences sampled from gorillas (accession numbers: FJ424863, FJ424864, FJ424865, FJ424866, FJ424871) were included to be used as an outgroup, and evolutionary analysis was performed in a Bayesian framework using BEAST [44]. A GTR+Γ model allowing for invariant rates among sites was used, with a log-normal relaxed molecular clock model and analysis performed using various tree priors, including constant size, exponential growth, logistic growth and Bayesian Skyline. Bayesian analysis was calibrated by setting priors on the tip nodes of the tree, referring to the year in which sequences were sampled. As a measure of reliability between the different tree priors, Bayes' factors were calculated, representing the ratio of the marginal likelihood between each of the pairs of models. Additionally, for Bayesian skyline analysis, effective population size growth rate was estimated over time, for the tree as a whole, as well as for selected clusters in the tree, and visualised using Bayesian skyline plots in Tracer.

#### Estimation of selection pressure

The ratio of non-synonymous versus synonymous mutations (dN/dS) was estimated with HyPhy [45] on the 190 concatenated sequences, with the protease and reverse transcriptase codons involved in ARV resistance removed. The ML phylogenetic tree was divided into two partitions (H and T strains), and estimation of the dN/dS ratios of the resulting clusters was performed by running the SelectionLRT analysis with a GTR model.

## Supporting Information

S1 Text Genetic distance. Comparison of mean group O and group M genetic distances observed intra- and inter-subgroups.
(DOC)

S2 Text Phylogenetic exploration. Evaluation of the previous classifications, and distribution of the strains depending on their sampling time and location.
(DOC)

S3 Text Recombination analyses. Evaluation of the recombination signal in our dataset.
(DOC)

S1 Fig Mean group O and group M genetic distances, intra- and inter- subgroups. The mean pairwise uncorrected p-distances observed within each group O subgroup (black) was compared to that observed in each group M subtype (light grey), as well as the mean intra-subtype and the mean inter-subtype distances observed for group M (dark grey).
(PDF)

S2 Fig Phylogenetic analysis of HIV sequences. **a)** Maximum likelihood tree inferred from the 190 concatenated group O sequences, with bootstrap values >70 (same tree as main text Fig 1) and colours highlighting the previous nomenclature from [18]: Blue = clade A (N = 146); Red = clade B (N = 7); Green = clade C (N = 10); Black = not classified (N = 26). **b)** Same tree as (a) with colours highlighting the previous nomenclature from [19]: Blue = cluster I (N = 111); Red = cluster II (N = 7); Green = cluster III (N = 10); Yellow = cluster IV (N = 7); Pink = cluster V (N = 4); Black = not classified (N = 51). Due to the partial sequences available from [19], it was not possible to include them in the concatenated alignment; the identification of the clusters was thus made using a env gp41 tree involving our strains and those from [19], see S3 Fig. **c)** same tree as (a), with colours highlighting the sampling country: Blue = France (N = 102); Red = Cameroon (N = 87): Green = Gabon (N = 1). **d)** Same tree as (a) with colours highlighting the time of sampling: Blue = 1987–1997 (N = 38); Green = 1997–2002 (N = 39); Orange = 2003–2007 (N = 38); Red = 2007–2012 (N = 39); Grey = ND or different sampling time in the different regions (N = 36).
(PDF)

S3 Fig Phylogenetic analysis of 360 HIV-1 Group O partial gp41 sequences (513 nucleotides). Maximum likelihood tree inferred using MEGA 5.0 with a GTR+Γ+I model; 1000 Bootstrap replicates were performed, and bootstrap values higher than 70% are indicated. Symbols highlight the sequences previously included in [19] and the cluster they were assigned to: Blue = cluster I (triangle: subcluster Ia, round: subcluster Ib, square: subcluster Iu); Red = cluster II; Green = cluster III; Yellow = cluster IV; Pink = cluster V; Black: unclassified.
(PDF)

S4 Fig Phylogenetic analysis of HIV-1 group O individual region sequences. **a)** Maximum likelihood tree inferred from the 190 protease and partial Reverse Transcriptase group O sequences, with colours highlighting the previous nomenclature from [18]: Blue = clade A (N = 146); Red = clade B (N = 7); Green = clade C (N = 10); Black = not classified (N = 26). Sequences belonging to population H are indicated. **b)** Same tree as (a) from the 190 integrase sequences. **c)** same tree as (a), from the 190 gp41 sequences.
(PDF)

## References

[1] Mourez T, Simon F, Plantier JC (2013) Non-M variants of human immunodeficiency virus type 1. Clin Microbiol Rev 26: 448–461. 10.1128/CMR.00012-13 23824367 PMC3719493

[2] Sharp PM, Hahn BH (2011) Origins of HIV and the AIDS Pandemic. Cold Spring Harb Perspect Med 1: a006841 10.1101/cshperspect.a006841 22229120 PMC3234451

[3] D'Arc M, Ayouba A, Esteban A, Learn GH, Boue V, et al (2015) Origin of the HIV-1 group O epidemic in western lowland gorillas. Proc Natl Acad Sci U S A 112: E1343–1352. 10.1073/pnas.1502022112 25733890 PMC4371950

[4] Worobey M, Gemmel M, Teuwen DE, Haselkorn T, Kunstman K, et al (2008) Direct evidence of extensive diversity of HIV-1 in Kinshasa by 1960. Nature 455: 661–664. 10.1038/nature07390 18833279 PMC3682493

[5] Faria NR, Rambaut A, Suchard MA, Baele G, Bedford T, et al (2014) HIV epidemiology. The early spread and epidemic ignition of HIV-1 in human populations. Science 346: 56–61. 10.1126/science.1256739 25278604 PMC4254776

[6] Lemey P, Pybus OG, Rambaut A, Drummond AJ, Robertson DL, et al (2004) The molecular population genetics of HIV-1 group O. Genetics 167: 1059–1068. 15280223 10.1534/genetics.104.026666 PMC1470933

[7] Vessiere A, Rousset D, Kfutwah A, Leoz M, Depatureaux A, et al (2010) Diagnosis and Monitoring of HIV-1 Group O-Infected Patients in Cameroun. J Acquir Immune Defic Syndr 53: 107–110. 10.1097/QAI.0b013e3181b97ec1 19770803

[8] Ayouba A, Mauclere P, Martin PM, Cunin P, Mfoupouendoun J, et al (2001) HIV-1 group O infection in Cameroon, 1986 to 1998. Emerg Infect Dis 7: 466–467. 11384531 10.3201/eid0703.010321 PMC2631804

[9] Vergne L, Bourgeois A, Mpoudi-Ngole E, Mougnutou R, Mbuagbaw J, et al (2003) Biological and genetic characteristics of HIV infections in Cameroon reveals dual group M and O infections and a correlation between SI-inducing phenotype of the predominant CRF02_AG variant and disease stage. Virology 310: 254–266. 12781713 10.1016/s0042-6822(03)00167-3

[10] Depatureaux A, Leoz M, De Oliveira F, Gueudin M, Damond F, et al (2010) [Specific diagnosis and follow-up of HIV-1 group O infection: RES-O data]. Med Mal Infect 40: 669–676. 10.1016/j.medmal.2010.04.011 20646884

[11] Arien KK, Abraha A, Quinones-Mateu ME, Kestens L, Vanham G, et al (2005) The replicative fitness of primary human immunodeficiency virus type 1 (HIV-1) group M, HIV-1 group O, and HIV-2 isolates. J Virol 79: 8979–8990. 15994792 10.1128/JVI.79.14.8979-8990.2005 PMC1168791

[12] Geuenich S, Kaderali L, Allespach I, Sertel S, Keppler OT (2009) Biological signature characteristics of primary isolates from human immunodeficiency virus type 1 group O in ex vivo human tonsil histocultures. J Virol 83: 10494–10503. 10.1128/JVI.00928-09 19706709 PMC2753123

[13] Vigan R, Neil SJ (2011) Separable determinants of subcellular localization and interaction account for the inability of group O HIV-1 Vpu to counteract tetherin. J Virol 85: 9737–9748. 10.1128/JVI.00479-11 21775465 PMC3196455

[14] Sauter D, Schindler M, Specht A, Landford WN, Munch J, et al (2009) Tetherin-driven adaptation of Vpu and Nef function and the evolution of pandemic and nonpandemic HIV-1 strains. Cell Host Microbe 6: 409–421. 10.1016/j.chom.2009.10.004 19917496 PMC2779047

[15] Ratner L, Haseltine W, Patarca R, Livak KJ, Starcich B, et al (1985) Complete nucleotide sequence of the AIDS virus, HTLV-III. Nature 313: 277–284. 2578615 10.1038/313277a0

[16] Vanden Haesevelde M, Decourt JL, De Leys RJ, Vanderborght B, van der Groen G, et al (1994) Genomic cloning and complete sequence analysis of a highly divergent African human immunodeficiency virus isolate. J Virol 68: 1586–1596. 8107220 10.1128/jvi.68.3.1586-1596.1994 PMC236616

[17] Quinones-Mateu ME, Albright JL, Mas A, Soriano V, Arts EJ (1998) Analysis of pol gene heterogeneity, viral quasispecies, and drug resistance in individuals infected with group O strains of human immunodeficiency virus type 1. J Virol 72: 9002–9015. 9765445 10.1128/jvi.72.11.9002-9015.1998 PMC110317

[18] Roques P, Robertson DL, Souquiere S, Damond F, Ayouba A, et al (2002) Phylogenetic analysis of 49 newly derived HIV-1 group O strains: high viral diversity but no group M-like subtype structure. Virology 302: 259–273. 12441070 10.1006/viro.2002.1430

[19] Yamaguchi J, Vallari AS, Swanson P, Bodelle P, Kaptue L, et al (2002) Evaluation of HIV type 1 group O isolates: identification of five phylogenetic clusters. AIDS Res Hum Retroviruses 18: 269–282. 11860674 10.1089/088922202753472847

[20] Tebit DM, Lobritz M, Lalonde M, Immonen T, Singh K, et al (2010) Divergent evolution in reverse transcriptase (RT) of HIV-1 group O and M lineages: impact on structure, fitness, and sensitivity to nonnucleoside RT inhibitors. J Virol 84: 9817–9830. 10.1128/JVI.00991-10 20631150 PMC2937803

[21] Agut H, Candotti D, Rabanel B, Huraux JM, Remy G, et al (1992) Isolation of atypical HIV-1-related retrovirus from AIDS patient. Lancet 340: 681–682. 10.1016/0140-6736(92)92226-6 1355252

[22] Faria NR, Suchard MA, Abecasis A, Sousa JD, Ndembi N, et al (2012) Phylodynamics of the HIV-1 CRF02_AG clade in Cameroon. Infect Genet Evol 12: 453–460. 10.1016/j.meegid.2011.04.028 21565285 PMC4677783

[23] Njouom R, Nerrienet E, Dubois M, Lachenal G, Rousset D, et al (2007) The hepatitis C virus epidemic in Cameroon: genetic evidence for rapid transmission between 1920 and 1960. Infect Genet Evol 7: 361–367. 17137845 10.1016/j.meegid.2006.10.003

[24] Nerrienet E, Pouillot R, Lachenal G, Njouom R, Mfoupouendoun J, et al (2005) Hepatitis C virus infection in cameroon: A cohort-effect. J Med Virol 76: 208–214. 15834878 10.1002/jmv.20343

[25] Pepin J, Labbe AC (2008) Noble goals, unforeseen consequences: control of tropical diseases in colonial Central Africa and the iatrogenic transmission of blood-borne viruses. Trop Med Int Health 13: 744–753. 10.1111/j.1365-3156.2008.02060.x 18397182

[26] Lachenal G (2014) Le médicament qui devait sauver l'Afrique. Paris: Editions La Découverte.

[27] Nyamweya S, Hegedus A, Jaye A, Rowland-Jones S, Flanagan KL, et al (2013) Comparing HIV-1 and HIV-2 infection: Lessons for viral immunopathogenesis. Rev Med Virol 23: 221–240. 10.1002/rmv.1739 23444290

[28] Archer J, Robertson DL (2007) Understanding the diversification of HIV-1 groups M and O. Aids 21: 1693–1700. 17690566 10.1097/QAD.0b013e32825eabd0

[29] Pepin J, Lavoie M, Pybus OG, Pouillot R, Foupouapouognigni Y, et al (2010) Risk factors for hepatitis C virus transmission in colonial Cameroon. Clin Infect Dis 51: 768–776. 10.1086/656233 20735242

[30] Drucker E, Alcabes PG, Marx PA (2001) The injection century: massive unsterile injections and the emergence of human pathogens. Lancet 358: 1989–1992. 11747942 10.1016/S0140-6736(01)06967-7

[31] Pepin J, Labbe AC, Mamadou-Yaya F, Mbelesso P, Mbadingai S, et al (2010) Iatrogenic transmission of human T cell lymphotropic virus type 1 and hepatitis C virus through parenteral treatment and chemoprophylaxis of sleeping sickness in colonial Equatorial Africa. Clin Infect Dis 51: 777–784. 10.1086/656232 20735238

[32] Pépin J (2011) The origins of AIDS. Cambridge: Cambridge University Press.

[33] Jonassen TO, Stene-Johansen K, Berg ES, Hungnes O, Lindboe CF, et al (1997) Sequence analysis of HIV-1 group O from Norwegian patients infected in the 1960s. Virology 231: 43–47. 9143301 10.1006/viro.1997.8510

[34] Hooper E (1997) Sailors and star-bursts, and the arrival of HIV. BMJ 315: 1689–1691. 9448543 10.1136/bmj.315.7123.1689 PMC2128008

[35] Franqueville A (1984) Yaounde: construire une capitale.

[36] Gouellain R (1975) Douala, ville et histoire; Paris: Institut d'Ethnologie MdlH, editor.

[37] Nasah BT, Nguematcha R, Eyong M, Godwin S (1980) Gonorrhea, Trichomonas and Candida among gravid and nongravid women in cameroon. Int J Gynaecol Obstet 18: 48–52. 6106601 10.1002/j.1879-3479.1980.tb00240.x

[38] Kengne Fodouop Athanase B (2000) Un demi siècle de recherche urbaine au Cameroun. Réseau Inter-Africain d'Etudes Urbaines au Cameroun (RIEUCAM). Yaounde: Presses Universitaires de Yaounde.

[39] de Silva TI, van Tienen C, Onyango C, Jabang A, Vincent T, et al (2013) Population dynamics of HIV-2 in rural West Africa: comparison with HIV-1 and ongoing transmission at the heart of the epidemic. AIDS 27: 125–134. 10.1097/QAD.0b013e32835ab12c 23032414

[40] Galimand J, Frange P, Rouzioux C, Deveau C, Avettand-Fenoel V, et al (2010) Short communication: evidence of HIV type 1 complex and second generation recombinant strains among patients infected in 1997–2007 in France: ANRS CO06 PRIMO Cohort. AIDS Res Hum Retroviruses 26: 645–651. 10.1089/aid.2009.0201 20560794

[41] Leoz M, Depatureaux A, Vessiere A, Roquebert B, Damond F, et al (2008) Integrase polymorphism and HIV-1 group O diversity. Aids 22: 1239–1243. 10.1097/QAD.0b013e3283021c30 18525277

[42] Tamura K, Peterson D, Peterson N, Stecher G, Nei M, et al (2011) MEGA5: molecular evolutionary genetics analysis using maximum likelihood, evolutionary distance, and maximum parsimony methods. Mol Biol Evol 28: 2731–2739. 10.1093/molbev/msr121 21546353 PMC3203626

[43] Stamatakis A (2014) RAxML version 8: a tool for phylogenetic analysis and post-analysis of large phylogenies. Bioinformatics 30: 1312–1313. 10.1093/bioinformatics/btu033 24451623 PMC3998144

[44] Drummond AJ, Rambaut A (2007) BEAST: Bayesian evolutionary analysis by sampling trees. BMC Evol Biol 7: 214 17996036 10.1186/1471-2148-7-214 PMC2247476

[45] Pond SL, Frost SD, Muse SV (2005) HyPhy: hypothesis testing using phylogenies. Bioinformatics 21: 676–679. 15509596 10.1093/bioinformatics/bti079

