# Supplementary material for: The Two-Phase Emergence of Non Pandemic HIV-1 Group O in Cameroon
Source: PLoS Pathog. 2015 Aug 4;11(8):e1005029. doi: 10.1371/journal.ppat.1005029 (PMC4524642; doi:10.1371/journal.ppat.1005029)
Supplement: S1 Text — Comparison of mean group O and group M genetic distances observed intra- and inter-subgroups. (DOC) [file ppat.1005029.s001.doc]

**S1: GENETIC DISTANCES**

**S1 Methods**

Mean uncorrected p-distances were calculated using MEGA 5.0 on the 190 group O concatenated sequence alignment described above, as well as on an alignment of 190 group M sequences encompassing the same regions. Group M sequences were selected from the available near full length genomes from the LANL HIV database (<http://www.hiv.lanl.gov/>), to represent only “pure” subtypes, with no recombinant strains and only one sub-subtype represented when several existed so as not to under-estimate inter subtype genetic distances. All full-length sequences available for subtypes F1, H, J, K, were included, and all but one for subtype G (due to misalignment causing indels and frameshifts). For subtypes A1, B, C and D, sequences were selected so as to represent at least one for every country of sampling available, resulting in a concatenated alignment including A1 (N=27), B (N=33), C (N=33), D (N=29), F1 (N=24), G (N=34), H (N=4), J (N=4) and K (N=2) sequences.

**S1 Results**

The mean genetic distance observed between the H strains was very close to that found within group M subtypes, while the distance between T strains was closer to that observed between group M subtypes (S1 Fig 1). This confirmed the apparent branch length discrepancies between the "comet head" and the "comet tail". The mean genetic distance between H strains being significantly lower than the one observed between T strains (Student T test: p<10E-5) was also consistent with the H strains recent ancestry when compared to the global groups M and O emergence.

**S1 Figure. Mean group O and group M genetic distances, intra- and inter- subgroups.** The mean pairwise uncorrected p-distances observed within each group O subgroup (black) was compared to that observed in each group M subtype (light grey), as well as the mean intra-subtype and the mean inter-subtype distances observed for group M (dark grey).
