# Supplementary material for: The Two-Phase Emergence of Non Pandemic HIV-1 Group O in Cameroon
Source: PLoS Pathog. 2015 Aug 4;11(8):e1005029. doi: 10.1371/journal.ppat.1005029 (PMC4524642; doi:10.1371/journal.ppat.1005029)
Supplement: S2 Text — Evaluation of the previous classifications, and distribution of the strains depending on their sampling time and location. (DOC) [file ppat.1005029.s002.doc]

**S2: PHYLOGENETIC EXPLORATION**

**S2 Results**

Using the previous classifications from Roques and Yamaguchi on the Maximum Likelihood tree shown in main text Fig 1, we showed that these previous nomenclature systems were partially discordant and failed to describe the whole genetic diversity of HIV-1 group O. However they already showed the existence of a major population including 112 to 147 of our strains (58.9% to 77.3%) depending on the nomenclature used. H strains correspond to the previously described major clade A as defined in and (S2 Fig a), and include the major cluster I as defined in (S2 Fig b) or C181 in (main text Fig 1a). Some subclusters are observed in population H (H1, H2, H3, respectively corresponding to A1/Ib, A2/IV, and A3/unclassified according to the previous nomenclatures /) and T (T1 and T2 respectively corresponding to B/II and C/III).

**S2 Figure. Phylogenetic analysis of HIV sequences**. **a)** Maximum likelihood tree inferred from the 190 concatenated group O sequences, with bootstrap values >70 (same tree as main text Fig 1) and colours highlighting the previous nomenclature from : Blue = clade A (N=146); Red = clade B (N=7); Green = clade C (N=10); Black = not classified (N=26). **b)** Same tree as (a) with colours highlighting the previous nomenclature from : Blue = cluster I (N=111); Red = cluster II (N=7); Green = cluster III (N=10); Yellow = cluster IV (N=7); Pink = cluster V (N=4); Black = not classified (N=51). Due to the partial sequences available from , it was not possible to include them in the concatenated alignment; the identification of the clusters was thus made using a env gp41 tree involving our strains and those from , see S3 Fig. **c)** same tree as (a), with colours highlighting the sampling country: Blue = France (N=102); Red = Cameroon (N=87): Green = Gabon (N=1). **d)** Same tree as (a) with colours highlighting the time of sampling: Blue = 1987 – 1997 (N=38); Green = 1997 – 2002 (N=39); Orange = 2003 – 2007 (N=38); Red = 2007 – 2012 (N=39); Grey = ND or different sampling time in the different regions (N=36).

**S3 Figure. Phylogenetic analysis of 360 HIV-1 Group O partial gp41 sequences (513 nucleotides)**. Maximum likelihood tree inferred using MEGA 5.0 with a GTR+Γ+I model; 1000 Bootstrap replicates were performed, and bootstrap values higher than 70% are indicated. Symbols highlight the sequences previously included in and the cluster they were assigned to: Blue = cluster I (triangle: subcluster Ia, round: subcluster Ib, square: subcluster Iu); Red = cluster II; Green = cluster III; Yellow = cluster IV; Pink = cluster V; Black: unclassified.

More than half of the strains sequenced (102/190, 53.7%) were sampled from patients living in France, most of them with links to Cameroon. The ML tree (S2 Fig c) shows that strains from France were phylogenetically interspersed with those sampled in Cameroon, with no evidence of major clusters involving only strains sampled in France. The partitioning of the strains into the H and T subgroups was statistically independent from the country of sampling, with 78.4% of H strains in France and 75.9% in Cameroon (Chi-square test: p > 0.99).

We investigated a possible link between phylogenetic position and sampling time, using the 154 sequences for which all three gene fragments were obtained from a single sample of known sampling time. We defined four quartiles depending on the sampling year: from 1987 to 1997 (N=38), 1997 to 2002 (N=39), 2003 to 2007 (N=38), and 2007 to 2012 (N=39). The different sampling time categories were all globally represented across the tree (S2 Fig d), with only one small cluster involving recent sequences only (6 strains sampled from 2006 to 2011, 3 in France and 3 in Cameroon). All the quartiles were represented in each of the subgroups H and T, showing no association of one of these populations with a particular period of sampling: both alike could be sampled as early as in the 1990s and as recently as in the 2010s.
