# Supplementary material for: The Two-Phase Emergence of Non Pandemic HIV-1 Group O in Cameroon
Source: PLoS Pathog. 2015 Aug 4;11(8):e1005029. doi: 10.1371/journal.ppat.1005029 (PMC4524642; doi:10.1371/journal.ppat.1005029)
Supplement: S3 Text — Evaluation of the recombination signal in our dataset. (DOC) [file ppat.1005029.s003.doc]

**S3: RECOMBINATION ANALYSES**

**S3 Methods**

To rule out the possibility of recombinant sequences that may affect phylogenetic analysis, recombination was screened for using SplitsTree , Recco and GARD . SplitsTree networks were inspected for the three individual gene segments, and a Phi test for recombination, implemented in SplitsTree, was used to determine a p-value for within-gene recombination. The longer concatenated gene segments were screened for recombination using GARD (datamonkey.org) and Recco to determine recombination between the gene segments.

**S3 Results**

We investigated the tree structure in each of the three individual regions sequenced (pr-RT, integrase, gp41), and observed that the branching order of some subclusters could vary (S4 Fig), but without disrupting the head/tail subgroupings. No readily identifiable recombination structure was found that could explain the intra-head and intra-tail branching order fluctuations. Together with the bootstrap analysis, these results suggested that the phylogenetic signal was weak in each of the two populations, which can be due to most of the branches and clusters originating from deep in the tree structure. This is consistent with a model of geographically restricted exponential growth, even though the division between populations H and T remained and was strongly supported by bootstrap analysis (75%).

**S4 Figure. Phylogenetic analysis of HIV-1 group O individual region sequences**. **a)** Maximum likelihood tree inferred from the 190 protease and partial Reverse Transcriptase group O sequences, with colours highlighting the previous nomenclature from : Blue = clade A (N=146); Red = clade B (N=7); Green = clade C (N=10); Black = not classified (N=26). Sequencs belonging to population H are indicated. **b)** Same tree as (a) from the 190 integrase sequences. **c)** same tree as (a), from the 190 gp41 sequences.

1. Huson DH, Bryant D (2006) Application of phylogenetic networks in evolutionary studies. Mol Biol Evol 23: 254-267.

2. Maydt J, Lengauer T (2006) Recco: recombination analysis using cost optimization. Bioinformatics 22: 1064-1071.

3. Kosakovsky Pond SL, Posada D, Gravenor MB, Woelk CH, Frost SD (2006) Automated phylogenetic detection of recombination using a genetic algorithm. Mol Biol Evol 23: 1891-1901.

4. Roques P, Robertson DL, Souquiere S, Damond F, Ayouba A, et al. (2002) Phylogenetic analysis of 49 newly derived HIV-1 group O strains: high viral diversity but no group M-like subtype structure. Virology 302: 259-273.
