# Supplementary figures and images for: The Two-Phase Emergence of Non Pandemic HIV-1 Group O in Cameroon
Source: PLoS Pathog. 2015 Aug 4;11(8):e1005029. doi: 10.1371/journal.ppat.1005029 (PMC4524642; doi:10.1371/journal.ppat.1005029)

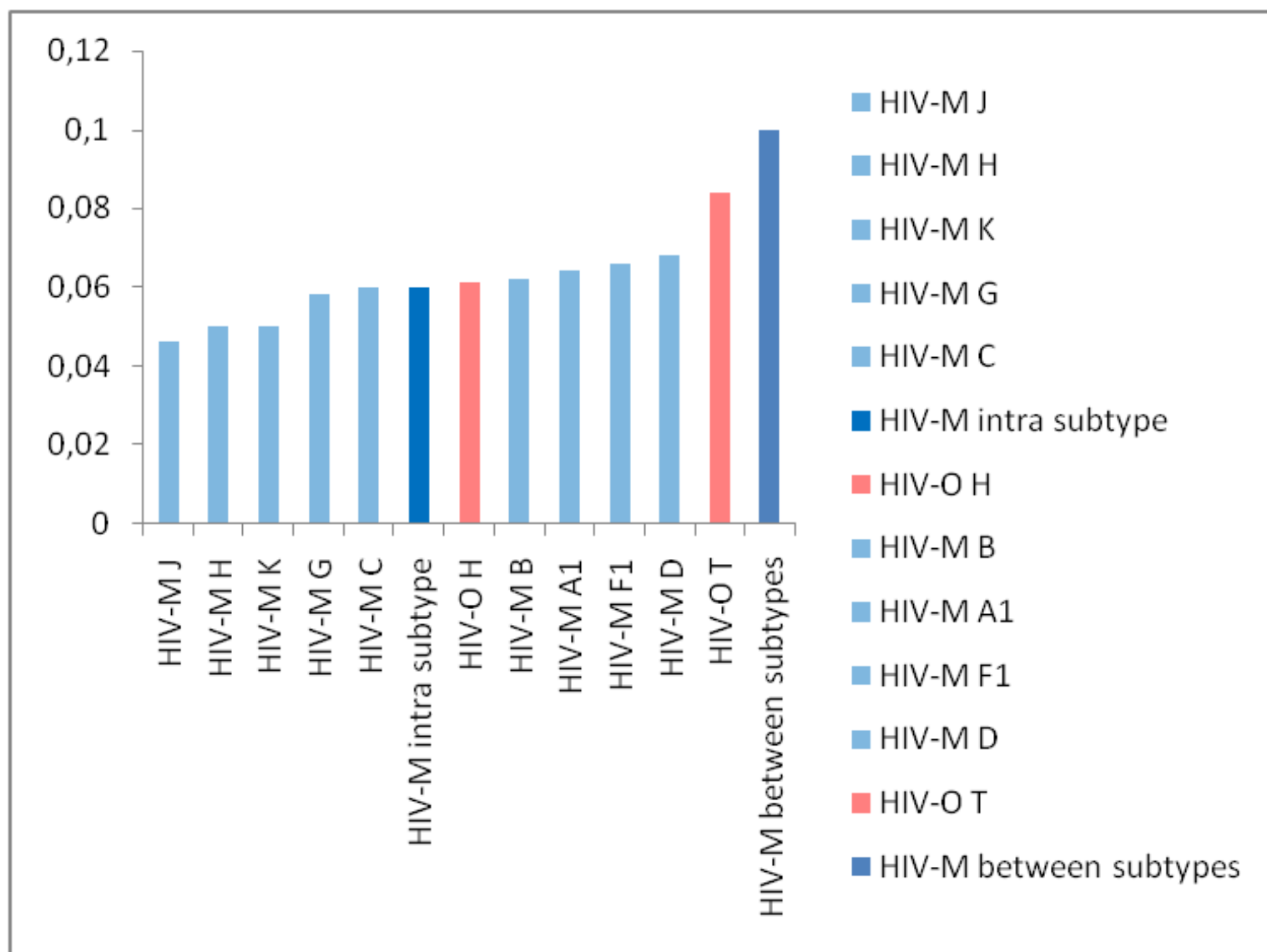

Supplement: S1 Fig — The mean pairwise uncorrected p-distances observed within each group O subgroup (black) was compared to that observed in each group M subtype (light grey), as well as the mean intra-subtype and the mean inter-subtype distances observed for group M (dark grey). (PDF) [file ppat.1005029.s004.pdf]

a)

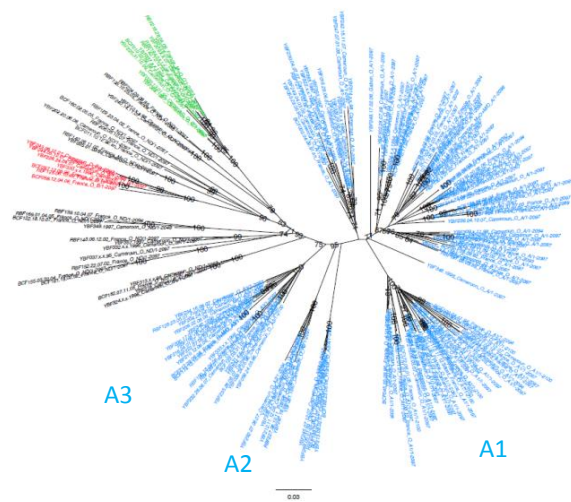

b)

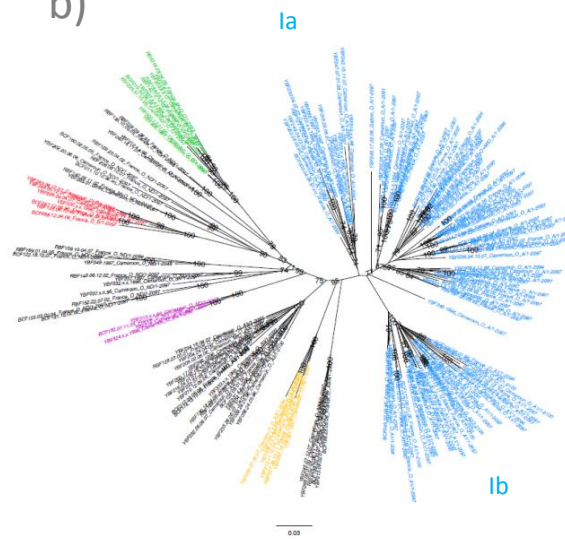

c)

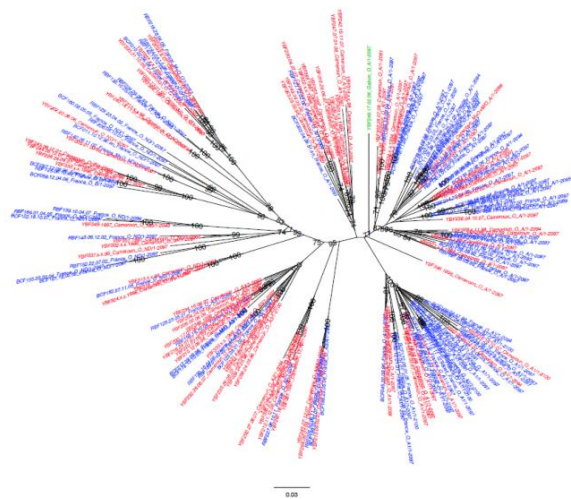

d)

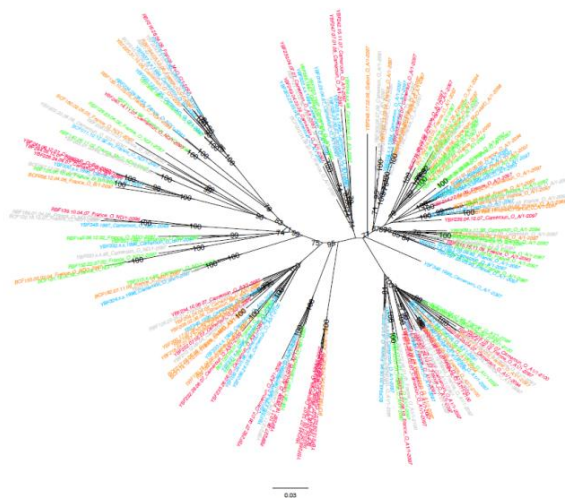

Supplement: S2 Fig — a) Maximum likelihood tree inferred from the 190 concatenated group O sequences, with bootstrap values >70 (same tree as main text Fig 1) and colours highlighting the previous nomenclature from [18]: Blue = clade A (N = 146); Red = clade B (N = 7); Green = clade C (N = 10); Black = not classified (N = 26). b) Same tree as (a) with colours highlighting the previous nomenclature from [19]: Blue = cluster I (N = 111); Red = cluster II (N = 7); Green = cluster III (N = 10); Yellow = cluster IV (N = 7); Pink = cluster V (N = 4); Black = not classified (N = 51). Due to the partial sequences available from [19], it was not possible to include them in the concatenated alignment; the identification of the clusters was thus made using a env gp41 tree involving our strains and those from [19], see S3 Fig. c) same tree as (a), with colours highlighting the sampling country: Blue = France (N = 102); Red = Cameroon (N = 87): Green = Gabon (N = 1). d) Same tree as (a) with colours highlighting the time of sampling: Blue = 1987–1997 (N = 38); Green = 1997–2002 (N = 39); Orange = 2003–2007 (N = 38); Red = 2007–2012 (N = 39); Grey = ND or different sampling time in the different regions (N = 36). (PDF) [file ppat.1005029.s005.pdf]

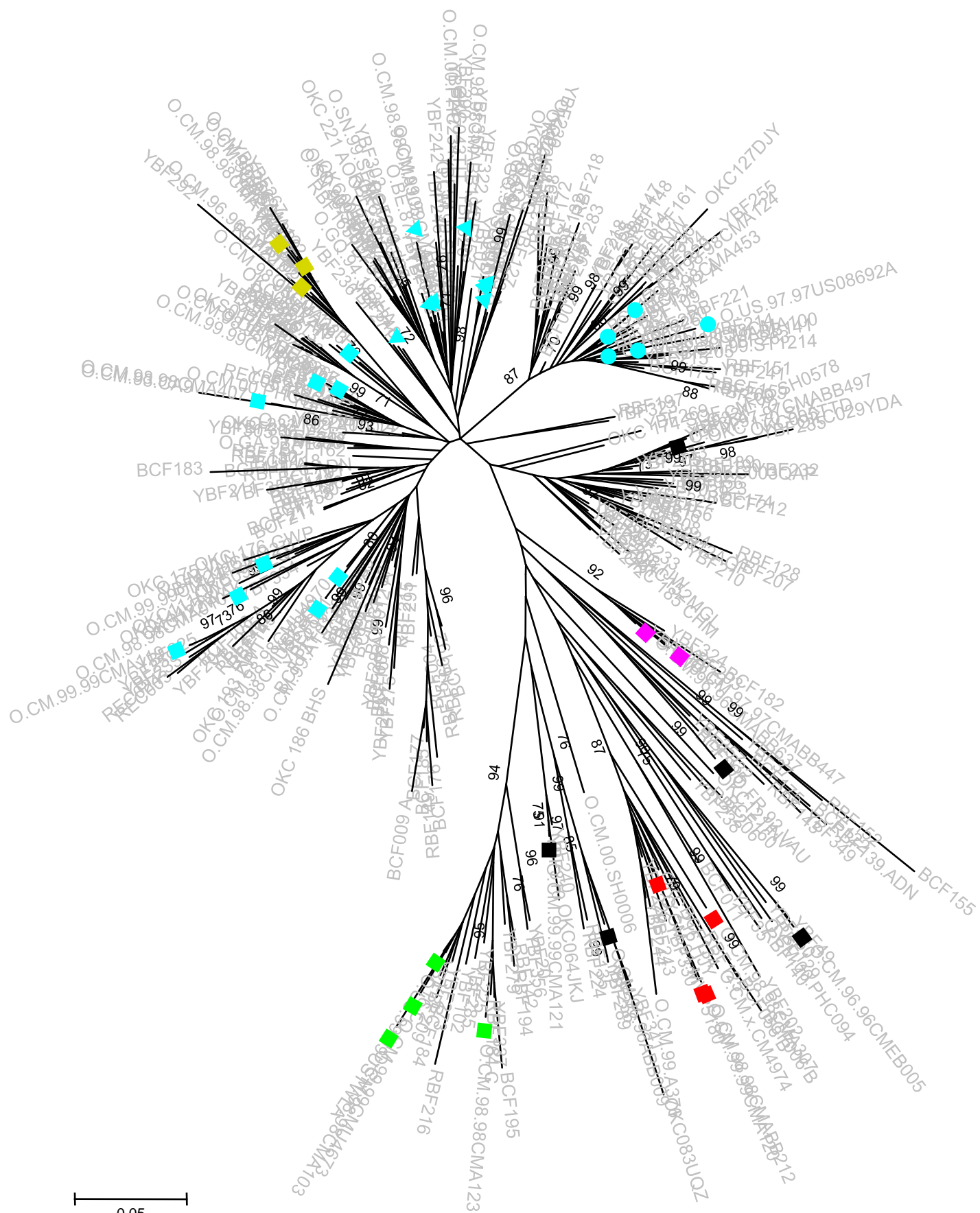

Supplement: S3 Fig — Maximum likelihood tree inferred using MEGA 5.0 with a GTR+Γ+I model; 1000 Bootstrap replicates were performed, and bootstrap values higher than 70% are indicated. Symbols highlight the sequences previously included in [19] and the cluster they were assigned to: Blue = cluster I (triangle: subcluster Ia, round: subcluster Ib, square: subcluster Iu); Red = cluster II; Green = cluster III; Yellow = cluster IV; Pink = cluster V; Black: unclassified. (PDF) [file ppat.1005029.s006.pdf]

a)

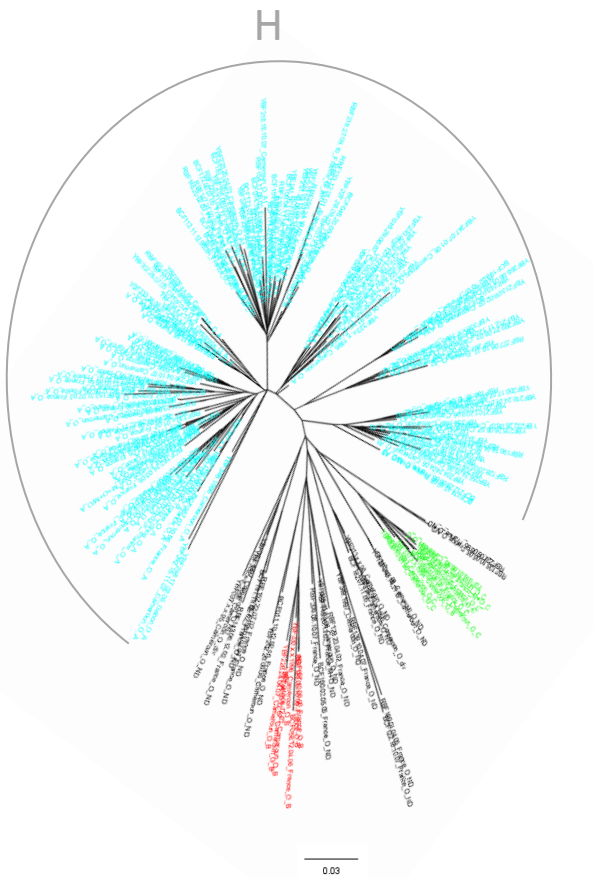

b)

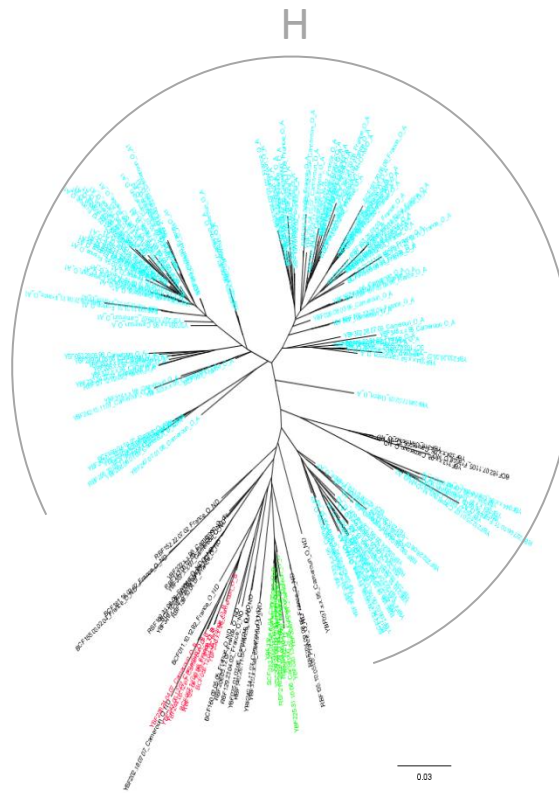

c)

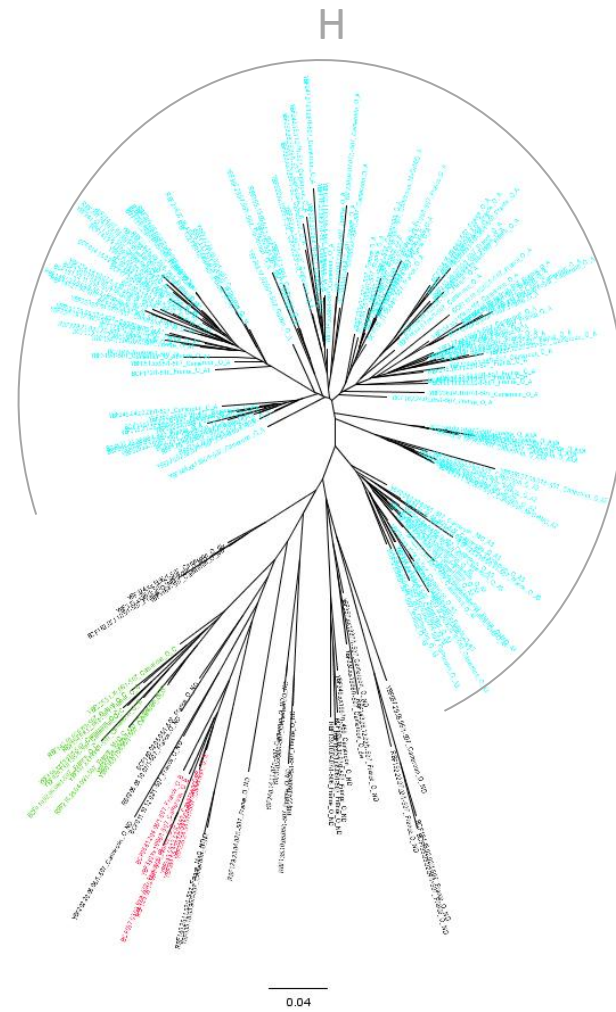

Supplement: S4 Fig — a) Maximum likelihood tree inferred from the 190 protease and partial Reverse Transcriptase group O sequences, with colours highlighting the previous nomenclature from [18]: Blue = clade A (N = 146); Red = clade B (N = 7); Green = clade C (N = 10); Black = not classified (N = 26). Sequences belonging to population H are indicated. b) Same tree as (a) from the 190 integrase sequences. c) same tree as (a), from the 190 gp41 sequences. (PDF) [file ppat.1005029.s007.pdf]
